# Short range magnetic exchange interaction favors ferroelectricity

**DOI:** 10.1038/srep22743

**Published:** 2016-03-09

**Authors:** Xiangang Wan, Hang-Chen Ding, Sergey Y. Savrasov, Chun-Gang Duan

**Affiliations:** 1 1National Laboratory of Solid State Microstructures, College of Physics, Nanjing University, Nanjing 210093, China; 2 Collaborative Innovation Center of Advanced Microstructures, Nanjing University, Nanjing 210093, China; 3 Key Laboratory of Polar Materials and Devices, Ministry of Education, East China Normal University, Shanghai, 200062, China; 4 Department of Physics, University of California, Davis, One Shields Avenue, Davis, CA 95616, USA

## Abstract

Multiferroics, where two or more ferroic order parameters coexist, is one of the hottest fields in condensed matter physics and materials science. To search multiferroics, currently most researches are focused on frustrated magnets, which usually have complicated magnetic structure and low magnetic ordering temperature. Here, we argue that actually simple interatomic magnetic exchange interaction already contains a driving force for ferroelectricity, thus providing a new microscopic mechanism for the coexistence and strong coupling between ferroelectricity and magnetism. We demonstrate this mechanism by showing that even the simplest antiferromagnetic insulator like MnO, could display a magnetically induced ferroelectricity under a biaxial strain. In addition, we show that such mechanism also exists in the most important single phase multiferroics, i.e. BiFeO_3_, suggesting that this mechanism is ubiquitous in systems with superexchange interaction.

The combination of different ferroic properties, especially ferroelectricity and (anti)ferromagnetism, provides additional degree of freedom to control magnetic and dielectric properties of the material. Such functionality is of great potential in applications to next-generation fast, portable and low-energy consumption data storage and processing devices^1^,^2^,^3^,^4^,^5^,^6^,^7^,^8^,^9^,^10^. Unfortunately, magnetism and ferroelectricity tend to be mutually exclusive, as conventional ferroelectric perovskite oxides usually require transition metal (TM) ions with a formal configuration *d*^0^, whereas magnetism, in contrast, needs TM ions with partially filled *d* shells^11^,^12^. As a consequence, simultaneous occurrence of magnetism and ferroelectricity is hard to be achieved, especially at room temperature. There are indeed some exceptions in Bismuth related magnetic oxides, *e.g.*, BiXO_3_ (X = Fe, Cr, Co, Mn)^8^,^13^. In these compounds, however, the magnetism and ferroelectricity are widely believed to have different origins, consequently the magnetoelectric coupling effects are rather weak^8^. Recently, dramatic progress on the Bismuth-based superstructures has been made and these structures could be very promising in realistic applications^14^,^15^,^16^.

To gain a strong magnetoelectric coupling, vast efforts have been devoted to search the improper ferroelectricity where electric dipoles are induced by magnetism^7^. Phenomenological theory suggests that spatial variation of magnetization is essential for the magnetically induced electric polarization^17^. Several microscopic mechanisms^18^,^19^,^20^, all emphasizing the importance of spin-orbital coupling, have also been proposed to explain the ferroelectricity in magnetic spiral structures. Electric polarization can also be induced by collinear spin order in the frustrated magnet with several species of magnetic ions^7^,^21^,^22^,^23^. In addition, it had been suggested that coupling between magnetic and charge ordering may results in ferroelectric magnets^2^. There are also proposals to realize multiferroic state in composite systems, or materials with nanoscale inhomogeneity^24^,^25^,^26^, or metal-organic hybrid systems^27^. Nevertheless, currently all of the known magnetically driven single-phase multiferroics require either Dzyaloshinskii-Moriya interaction (DMI)^28^,^29^, which is small in strength, or competing exchange interactions in real space. Therefore, they generally have complex magnetic order, low transition temperature (below several ten K) and small electric polarization (generally two to three orders of magnitude smaller than those of typical ferroelectrics), making them still far away from practical applications. Therefore searching new mechanism for multiferroicity is of both fundamental and technological interest.

In this study, we demonstrate that regardless the usually weak spin-orbit coupling, simple interatomic magnetic exchange interaction already provides a driving force to break the inversion symmetry of the system, which is necessary for the occurrence of ferroelectricity. It is therefore a new microscopic mechanism for the coexistence and strong coupling between ferroelectricity and magnetism. Using band structure calculations, we numerically confirm this new mechanism by illustrating that even the simplest antiferromagnets, i.e. MnO, can display a magnetically induced ferroelectricity under a strong biaxial strain. In addition, we show that such mechanism also exists in BiFeO_3_ (BFO).

To study the interatomic magnetic exchange interaction and the associated magnetic ordering energy, we consider a three atoms case, where a diamagnetic ion such as oxygen ion sits between two transition metal ions, as shown in Fig. 1. As revealed by Sergienlo and Dagotto^19^, DMI provides a driving force for the oxygen ion to shift perpendicularly to the spin chain. Due to the small strength, however, the energy gain from DMI is usually less than the ordinary elastic energy, consequently only a few compounds show magnetically induced ferroelectricity^7^. Here, we consider the effect of longitudinal displacement of diamagnetic ions (shown by the dot line in Fig. 1) on the magnetic ordering energy. As is well known, in the above case, the distance between magnetic ions usually is much larger than the radii of *d*/*f* orbital which carry magnetic moments, thus the direct exchange is negligible, and the hybridization between magnetic and diamagnetic ions is essential for the indirect magnetic exchange coupling. Therefore, we first discuss the hopping integrals in this system. A generalized Hamiltonian with explicitly written hopping integral between metal ions and anions is shown as follows:


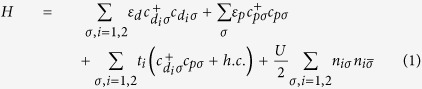


where the operator 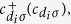 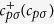 creates (annihilates) a spin *σ* electron at transition metal and O site, respectively. *ε*_*d*_ and *ε*_*p*_ are the onsite energies for M_1_/M_2_ and O site. *t*_*i*_ is the hopping integral between transition metal ion at site *i* and O. 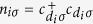
and *U* is the Coulomb repulsion energy. Based on the standard Schrieffer-Wolff transformation^30^, we can eliminate the O orbital and obtain the effective hopping integral between M_1_ and M_2_ site:


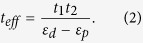


As is well known, the hopping integral is inversely proportional to the bond-length, i.e.,


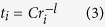


where *r*_*i*_ is the bond length of M_*i*_-O, *l* is a positive value and strongly depends on both the bond type and the participated orbital^31^. It is well known that a small change of bond length has only small effect on the on-site energy, thus basically the parameter of *C* in equation [3] is not sensitive to *r*_*i*_. A longitudinal displacement of O ion *u* will then change the effective hopping to


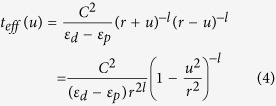


where *r* is the distance between magnetic ion and center O-site. It is interesting to notice that regardless the parameter *C* and the superscript *l* in equation [4], a displacement *u* which breaks inversion symmetry always increases the effective hopping between the magnetic ions.

To see the effect of *u* on the magnetic ordering energy, we take the superexchange, which is one of the most common mechanisms in magnetic insulators, as an example. It is well known that for superexchange in Mott-Hubbard insulators, the interatomic exchange interaction can be written as^32^:


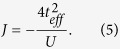


where *U* is the Coulomb interaction for the magnetic orbital. Consequentlly, an off-center displacement of O ion can enhance the interatomic exchange interaction. Usually the magnetic ordering energy has the form of 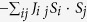, thus for the AFM case, the energy gain up to the second order of the longitudinal displacement of O ions *u* is


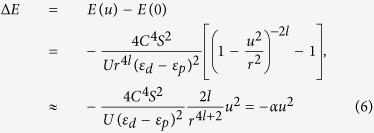


where


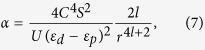


and *S* is the spin on the metal ion. Thus we prove that regardless the exact formula for the hopping dependence on distance, an off-center distortion can definitely lower the total energy by an amount proportional to *u*^2^.

Above we have discussed the superexchange in one-band case. For multi-band superexchange and even double exchange mechanism^33^, the exchange coupling *J* is also proportional to the effective hopping, just the relationship between *J* and *t*_*eff*_ in these mechanisms may not be as simple as shown in equation [5]. Keeping in mind that the effective hopping is a function of 1/(*r*^2^−*u*^2^) as shown in equation [4], an off-center distortion will therefore always lower the magnetic energy. Moreover, regardless the specific form of *J* (*t*_*eff*_), the corresponding energy gain is still ~*u*^2^, as in the case of the single band superexchange. Now we see that both the magnetic exchange energy change and the ordinary elastic energy change (~*Ku*^2^/2) are proportional to the square of the O off-center displacement, yet with different sign. Therefore, when the anion located between magnetic ions is shifted away from the center, the increase in elastic energy tends to be compensated by the decrease of magnetic exchange energy, and consequently, may form an electric dipole and resulting in ferroelectricity, as Fig. 1 shows.

The ferroelectricity is long believed to originate from a delicate balance between the short-range forces favoring the undistorted paraelectric structure and the long range Coulomb interactions favoring the ferroelectric phase^34^,^35^. Now we see that indirect magnetic exchange, which is short-range in nature, provide another driving force for the off-center atomic motion. To our knowledge, this demonstrates a new mechanism for ferroelectricity.

One may argue that though the indirect magnetic interaction will lower the total energy with off-center atomic movement, if the value of α is much smaller than the elastic constant *K*, the ferroelectricity may never have chance to appear. We, however, by both empirical and numerical calculations, show that α is of the same magnitude of *K*, and indirect magnetic exchange induced ferroelectricity indeed occurs under certain circumstances.

We first use the typical value for transition metal oxides as an example to estimate the magnitude of α in equation [7] and elastic constant *K*. As given in ref. 31, for typical transition metal oxide, *t*_*pd*_ = *V*_*pdσ*_ ~ 2 eV, *l* = 3.5, *ε*_*d*_ − *ε*_*p*_ ~ 3 eV, *U*_d_ ~ 7 eV, *r* ~ 2 Å, thus we have *α* ~ 450 meV/Å^2^. This magnitude, as we expect, is smaller than that of regular elastic energy of transition metal oxides. Taking the nonmagnetic perovskite oxide cubic SrTiO_3_ as an example, we move Ti ion along the *z* direction, and obtain the total energy change with respect to the paraelectric phase as ∆ = *βu*^*2*^ with *β* ~ 1050 meV/Å^2^. That is why ferroelectricity generally does not occur in magnetic materials. But we expect that in some cases when elastic energy weakens, e.g. under epitaxial strain, the magnetic interaction induced ferroelectricity have chance to occur.

To numerically prove the above conclusion, we then carried out a series of first-principles calculations. For the purpose of avoiding the complication due to complex magnetic order and achieving an unambiguous result, we choose the simple insulator MnO with rock-salt structure, which is AFM at low temperature (~120 K) and paramagnetic (PM) under normal conditions, as the demonstrating system. Particularly, Mn^2+^, in a pure ionic point of view, has magnetic moment ~5 *μ*_B_, which is favorable of increasing magnetic energy according to equation [7].

Although cannot deal with the high-temperature PM state of MnO, DFT + *U* scheme is adequate for the zero-temperature magnetically ordered insulating state^36^. Thus we utilize the DFT + *U* method to check whether the ferroelectricity can be induced by a biaxial strain, which had been shown as a powerful method to reduce the elastic energy^37^. Details of the calculation are shown in Methods.

To explicitly show the contribution of the magnetic interaction, we write the total energy as:


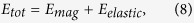


where *E*_mag_ is essentially the Heisenberg energy,


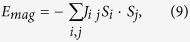


and the exchange constant *J*_*ij*_ can be obtained from energy fitting of different magnetic configurations. In the case of MnO which has rocksalt structure, due to the fact that the dominant magnetic interaction is between next-nearest neighbors^38^, we have


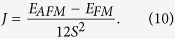


For cubic phase MnO with inversion center and choosing *U*_*eff*_  = 3 eV^39^, we obtain *J* ~ −1.4 meV. Monte Carlo (MC) simulations based on these parameters (see Methods), predict the Néel temperature to be 114 K, in good agreement with the experimental value ~120 K, supporting our exchange interaction by total energy calculations on MnO.

Then we consider the energy change due to O off-center displacement *u*. Here for simplicity we focus on the off-center (001)-direction motion of O ions. Our numerical results (see Fig. 2a) show that the magnetic energy change with *u* has negative sign and decreases quadratically with *u*, which perfectly agree with equation [6], as can be seen from the harmonic fitting (Δ*E* = −*αu*^*2*^) with the coefficient *α* ~ 207 meV/Å^2^ (the green straight lines in Fig. 2a). This magnitude is smaller than our estimated one possibly due to the much larger MnO bond (~2.2 Å), but it demonstrates that off-center motion of the anion between two magnetic cations would enhance the magnetic exchange interaction. To our knowledge, there has been no such report before. At strain-free state (0%), due to the stronger restoring force caused by the elastic energy change, the curve for total energy change with the O off-center displacement is parabolic (also see Fig. 2a), as a paraelectric state should be.

When the compressive strain applies, the *c*/*a* ratio increases, and the Mn-O bond along the *z* direction becomes larger. This will affect both the elastic energy and the magnetic energy. Indeed, *α* decreases to 189 (−4%) and 181 meV/Å^2^ (−5%). The elastic energy, however, is influenced more, as can be clearly seen from Fig. 2b,c. Consequently, the bottom of the total energy curve becomes flat and at a reasonable strain, e.g., −4% (Fig. 2b), ferroelectricity is induced in the ground state of MnO. At even larger compressive strain (−5%, Fig. 2c), the energy curve is clearly in the shape of double-well—a symbol of ferroelectricity. The so calculated polarization is about 0.38 C/m^2^, which is comparative to that of typical perovskite ferroelectrics. It’s worthy to mention that, in this scheme, as the occurrence of ferroelectricity is a result of the strengthened magnetic interactions, the antiferromagnetic *T*_N_ will be enhanced significantly due to the ferroelectric displacement according to equation [6]. For ferroelectric MnO under −5% strain, we estimate the *T*_N_ could be as large as 170 ~ 180 K.

To further check the validity of our theory, we carried out another set of calculation by adopting different Hubbard repulsing energies. Note that in our DFT + *U* calculation, the effective *U* parameter is not the same as the one used in analytical derivations. Nevertheless, their change should be the same. Our numerical results show that enlarging *U* will suppress the magnetic ordering energy, and consequently the critical strain for the occurrence of ferroelectricity will increase. Again, this is expected from our theory (see equation [5]), and demonstrates the importance of magnetic ordering energy for the ferroelectricity.

Speaking solely from the point of view of magnetic exchange interaction, antiferroelectric phases are also possible states to lower the magnetic energy of the system. We show here that these states are not energetically favored by considering the electrostatic energy. This is confirmed by our phonon frequency calculation on the paraelectric phase of a 2 × 2 × 2 supercell of MnO under different in-plane strains. We find that only the *A*_2u_ mode (vibration of Mn and O ion along *z*-axis) has been gradually softened when the compressive epitaxial strain increases. No lattice instability corresponds to an antiferroelectric phase. Therefore, the off-center O movement will result in ferroelectricity instead of antiferroelectricity.

To further confirm that the above ferroelectric instability is of magnetic origin instead of other mechanism^37^, we then perform LDA + DMFT calculation for the paramagnetic (PM) phase, i.e. high temperature phase of MnO (see Methods). All of our calculations, regardless the temperature (*T* = 200, 300 and 400 K) and *U*, give the same qualitative conclusion: losing the magnetic ordering will suppress the off-center displacement, and even a large strain (−5%) can no longer induce the ferroelectric instability for PM phase (see Supplementary Materials for details). Thus we unambiguously demonstrate that here the magnetic interaction is essential for the onset of ferroelectricity.

Finally, we have also carried out similar calculations on the most famous single phase multiferroics, i.e. BFO. Both the room-temperature rhombohedral (*R*) phase and strained tetragonal (*T*) phases are studied. Our results, as shown in Fig. 3, reveal that the magnetic energy is also decreasing quadratically with the O atom displacement along the neighboring Fe-Fe connection line when the movement is not large. What is more interesting is, in the *T*-BFO the contribution of the magnetic energy to the total energy decrease is much larger than that in the *R*-BFO. We attribute this to the enhanced superexchange interaction in T-phase, as in this phase Fe-O-Fe is exact 180 degree, whereas in *R* phase is about 165 degree, and according to the Goodenough-Kanamori rule^40^, 180 degree bond angle has the strongest superexchange interaction. This also explains the observed enhanced ferroelectric polarization in the T-BFO^41^.

In summary, we have confirmed both analytically and numerically that indirect magnetic exchange, contrary to what people previously thought, may favor ferroelectricity even in collinear magnetic systems. As shown in the above analysis, a bonus coming with the induced ferroelectricity would be the enhancing of the magnetic transition temperature. In addition, this mechanism is not confined in antiferromagnets, thus various magnetic systems could be potential multiferroics. Our research then provides a new way to explain the coexistence of ferroelectricity and magnetism and might be useful to the search of novel multiferroics suitable of practical use.

## Methods

### First-Principles Calculations

For the zero-temperature magnetically ordered insulating state, we use the projector augmented wave (PAW) method implemented in the Vienna Ab-Initio Simulation Package (VASP)^42^. The exchange-correlation potential is treated in the generalized gradient approximation (GGA). We use the energy cut-off of 500 eV for the plane wave expansion and a 10 × 10 × 10 Monkhorst-Pack grid for *k*-point sampling in the self-consistent calculations. The effective Hubbard constant *U*_eff_  = *U* − *J* from 1 to 6 eV is adopted to treat the strongly-correlated nature of MnO^43^. Variation of *U*_eff_ does not change our qualitative conclusions. In this study, the epitaxial strain is defined as (*a* − *a*_0_)/*a*_0_, where *a* is the in-plane lattice parameter and *a*_0_ is the theoretical equilibrium lattice constant in cubic symmetry. The out-of-plane lattice parameter *c* is optimized at every strain. The Berry phase technique is used to calculate ferroelectric polarizations^44^.

### Monte Carlo Simulation

Monte Carlo (MC) simulations, based on the model Heisenberg Hamiltonian with *ab initio* derived exchange parameters, are used to obtain the Néel temperature of MnO. The same method has been applied successfully in the study of rare-earth magnetic materials^45^. The lattice studied in our MC simulation is a 16a × 16a × 16*a* fcc cell (16384 spins) with periodic boundary conditions, where *a* is the lattice constant.

### LDA + DMFT method

We use the LDA + DMFT method^36^,^46^ to calculate the high temperature PM state. We use the highly accurate continue time quantum Monte Carlo (CT-QMC) as the impurity solver^46^ and cross check our results by the non-crossing approximation. Calculations are fully self-consistent in charge density, chemical potential, impurity level and total energy. For low temperature, CT-QMC would be very demanding on the computer resource, we thus only consider the high temperature PM phase of MnO by LDA + DMFT.

## Additional Information

**How to cite this article**: Wan, X. *et al.* Short range magnetic exchange interaction favors ferroelectricity. *Sci. Rep.* **6**, 22743; doi: 10.1038/srep22743 (2016).

## Supplementary Material

Supplementary Information

## Figures and Tables

**Figure 1:**
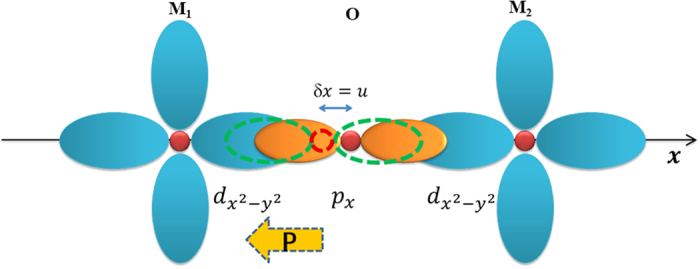
Illustration of ferroelectricity induced by indirect magnetic exchange interaction between anion-mediated magnetic cations. Here M_1_ and M_2_ are magnetic cations, O is oxygen anion. When O atom, which originally sits in the middle of M_1_ and M_2_ ions, is shifted along the M_1(2)_-O bond direction (*x*) by a small displacement *u*, the magnetic exchange interactions of the system will increase and may support the O off-center movement. An electric dipole is then formed, as shown by an arrow in the picture.

**Figure 2:**
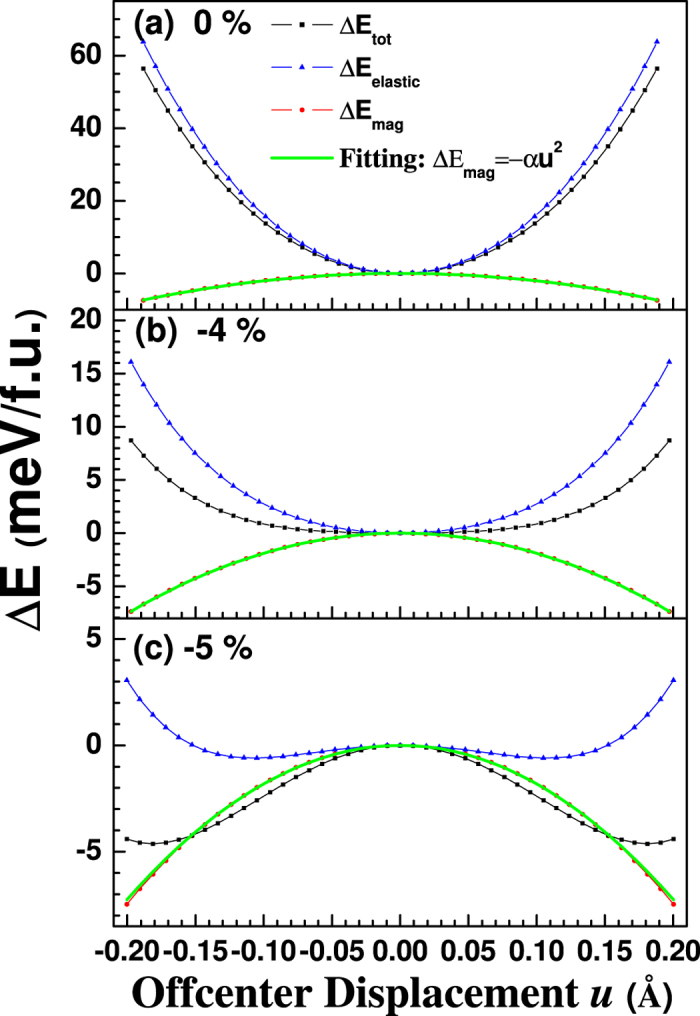
Calculated energy change with a series of O off-center displacement of MnO under different biaxial strains at zero-temperature: (**a**) no strain, (**b**) −4%, (**c**) −5%. Blue-triangles, red solid circles and black solid squares indicate the elastic, magnetic and total energies, respectively. Results come from DFT + *U* calculations with *U*_*eff*_  = 3.0 eV. Green straight lines are fitted curves according to ΔE = −α*u*^2^, which perfectly agree with the calculated ones. The obtained coefficient α for 0, −4% and −5% strain are 207, 189 and 181 meV/Å^2^, respectively.

**Figure 3:**
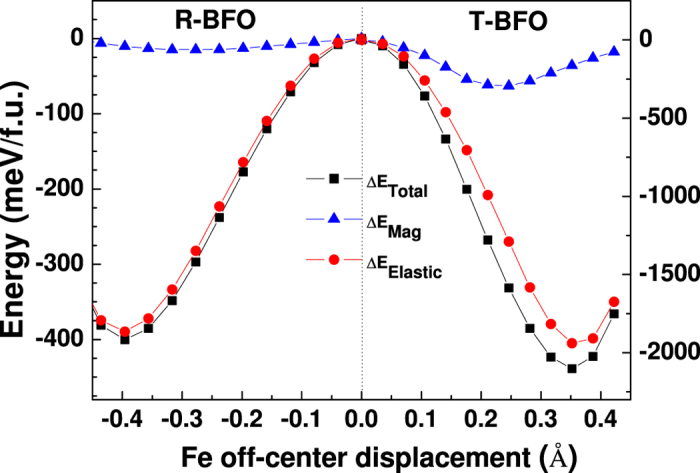
Calculated energy change with the O off-center displacement in R-BFO (left) and T-BFO (right). Note the different energy scale of the left and right panel. Blue-triangles, red solid circles and black solid squares indicate the elastic, magnetic and total energies, respectively.
