# Supplementary material for: Short range magnetic exchange interaction favors ferroelectricity
Source: Sci Rep. 2016 Mar 9;6:22743. doi: 10.1038/srep22743 (PMC4783704; doi:10.1038/srep22743)
Supplement: Supplementary Information [file srep22743-s1.doc]

Supplementary Information

Short range magnetic exchange interaction favors ferroelectricity

Xiangang Wan^a,1^, Hang-Chen Ding^b^, Sergey Y. Savrasov^c^, and Chun-Gang Duan^b,2^

^a^Department of Physics and National Laboratory of Solid State Microstructures, Nanjing University, Nanjing 210093, China; ^b^Key Laboratory of Polar Materials and Devices, Ministry of Education, East China Normal University, Shanghai 200062, China; ^c^Department of Physics, University of California, Davis, One Shields Avenue, Davis, CA 95616, USA

^1^xgwan@nju.edu.cn

^2^wxbdcg@gmail.com or cgduan@clpm.ecnu.edu.cn

**Paramagnetic calculation on strained MnO**

**
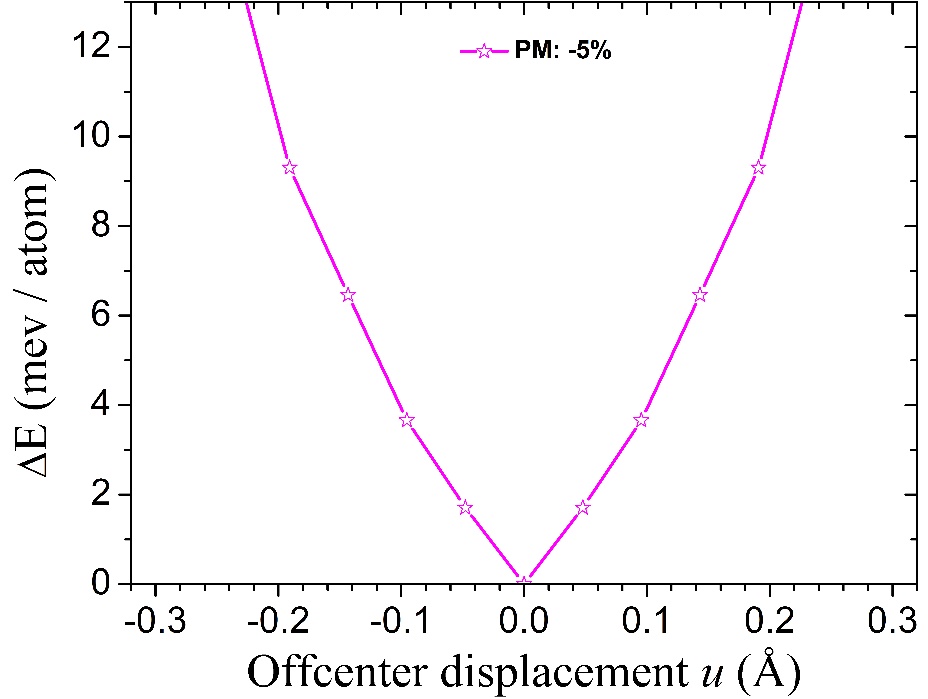
**

**Figure S.1| Energy difference as a function of O offcenter displacement for paramagnetic MnO.** The high-temperature result is from LDA+DMFT simulation with *U*=3.0 eV and T=300 K.
